# Supplementary material for: Pramipexole Hyperactivates the External Globus Pallidus and Impairs Decision-Making in a Mouse Model of Parkinson’s Disease
Source: Int J Mol Sci. 2024 Aug 14;25(16):8849. doi: 10.3390/ijms25168849 (PMC11354263; doi:10.3390/ijms25168849)
Supplement: Supplementary file 1 [file ijms-25-08849-s001.zip › ijms-3136915-supplementary.pdf]

Article

Pramipexole Hyperactivates the External Globus Pallidus and Impairs Decision-Making in a Mouse Model of Parkinson's Disease

Hisayoshi Kubota [1], Xinzhu Zhou [1], Xinjian Zhang [1], Hirohisa Watanabe [2] and Taku Nagai [1,*]

Supplementary Methods

Real-time reverse transcription-PCR

The striatum of each mouse was homogenized, and total RNA was extracted using the RNeasy Total RNA Isolation Kit (Qiagen, Hilden, NRW, Germany) and converted into cDNA using a ReverTra Ace Kit (Toyobo, Osaka, Japan). Quantitative real-time PCR was performed for D3R using SsoFast Probe SuperMix (BioRad, Hercules, CA, USA) with QuantStudio7 (Thermo Fisher Scientific, Waltham, MA, USA). The primers used were D3R (GenBank accession number NM007877) and β-actin (GenBank accession number NM007393). The reaction profile consisted of an initial round at 95°C for 30 s followed by 40 cycles of denaturation at 95°C for 5 s, and annealing at 60°C for 1 min in QuantStudio7. To standardize the quantification, β-actin was simultaneously calculated with D3R. The expression levels were calculated using the ΔΔCt method.

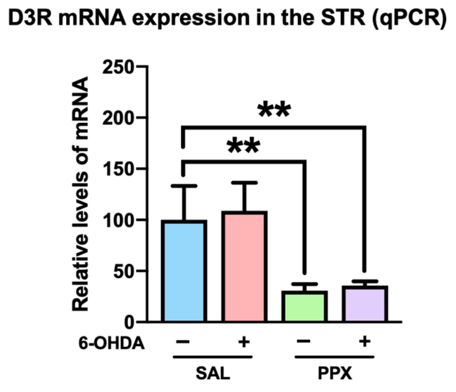

Figure S1. Expression of D3R in the STR of PPX-treated sham- and 6-OHDA-lesioned mice. Brain samples were collected on the last day of Period 1. mRNA levels of D3R in the STR were determined by quantitative PCR. The data were expressed as percentages of levels in sham-lesioned mice treated with saline. Each column represents the mean ± SEM (n = 8). **p < 0.01 versus Sham / SAL. STR, striatum; 6-OHDA, 6-hydroxydopamine; SAL, Saline; PPX, pramipexole; D3R, dopamine D3 receptor.

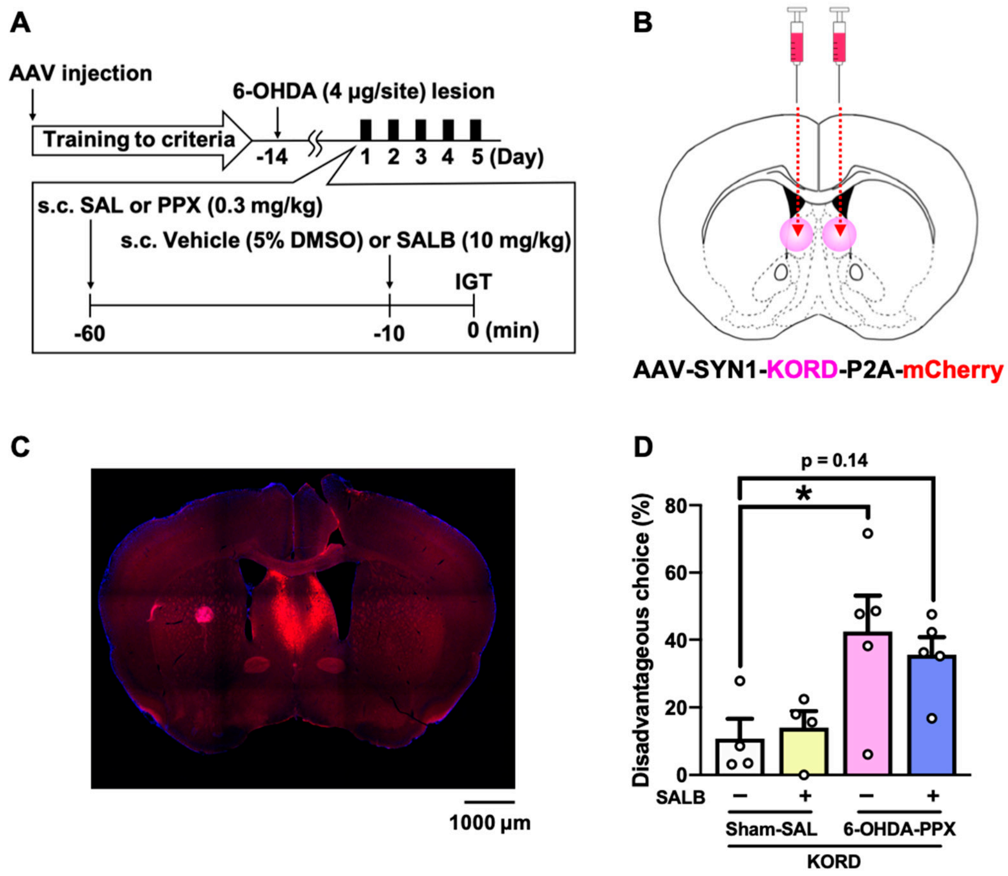

Figure S2. Effect of chemogenetic inhibition of the LS on PPX-induced decision-making impairments in 6-OHDA-lesioned mice. (A) Experimental schedule. Prior to IGT training, AAV-SYN1-KORD-P2A-mCherry was injected bilaterally into the LS. After meeting the criteria of IGT training, 6-OHDA-lesioned mice were co-administered PPX (0.3 mg/kg) and SALB (10 mg/kg) for 5 days, and their decision-making was assessed in the IGT. (B) The injection site of AAV-SYN1-KORD-P2A-mCherry. (C) Representative photograph of the expression of KORD-mCherry in the LS. Scale bar = 1000 μm. Decision-making was evaluated by percentage of (D) disadvantageous choices. Each column represents the mean ± SEM (n = 4~5). *$p < 0.05$ versus Sham-SAL / Vehicle. LS, lateral septum; KORD, kappa-opioid receptor-based DREADD; IGT, Iowa gambling task; 6-OHDA, 6-hydroxydopamine; SAL, Saline; PPX, pramipexole; SALB, salvinorin B.

Table S1. c-Fos mapping of 36 brain regions. Brain samples were collected 2 h after the IGT on the last day of Period 2. The number of c-Fos-positive cells in 36 brain regions was analyzed. Each line represents the mean ± SEM (n = 4~5). *p < 0.05, **p < 0.01 versus corresponding to SAL group. 6-OHDA, 6-hydroxydopamine; SAL, Saline; PPX, pramipexole; Cg, cingulate cortex; PrL, prelimbic cortex; IL, infralimbic cortex; MO, medial orbital cortex; VO, ventral orbital cortex; LO, lateral orbital cortex; NAc, nucleus accumbens; VP, ventral pallidum; LSD, lateral septum, dorsal part; LSI, lateral septum, intermediate part; LSV, lateral septum, ventral part; VTA, ventral tegmental area; SNr, substantia nigra pars reticulata; SNc, substantia nigra pars compacta; CA1/CA2/CA3; hippocampal subregions; DG, dentate gyrus; LP, lateral posterior nucleus; PV, paraventricular thalamic nucleus; LD, lateral dorsal nucleus; CL, central lateral nucleus; MD, mediodorsal nucleus; IMD, intermediodorsal nucleus; CM, central medial nucleus; PC, paracentral nucleus; Po, posterior complex; VPM, ventral posteromedial nucleus; Rt, reticular nucleus; VPL, ventral posterolateral nucleus; VM, ventral medial nucleus; STN, subthalamic nucleus; LH, lateral hypothalamus; PH, posterior hypothalamic nucleus; DMH, dorsomedial hypothalamic nucleus; VMH, ventromedial hypothalamic nucleus.

Brain region	Cg	PrL	IL	MO	VO	LO	NAc	VP	LSD
Sham/SAL	212.4±41.49	255.2±35.73	162.0±17.70	202.6±45.10	292.8±95.21	329.2±90.75	114.3±11.23	119.8±7.33	278.5±48.00
6-OHDA/SAL	159.6±33.24	183.4±25.38	142.9±37.14	151.4±45.00	253.1±57.96	275.0±55.95	156.2±29.44	158.7±26.67	241.7±21.65
Sham/PPX	244.6±64.36	265.8±69.19	168.3±37.15	161.6±49.68	262.2±81.52	295.1±66.24	101.4±13.89	114.3±14.66	360.9±67.67
6-OHDA/PPX	155.0±30.29	211.5±36.11	143.3±28.85	108.5±31.44	193.6±68.14	285.4±98.38	79.0±15.02	87.9±16.03	322.4±55.35

Brain region	LSI	LSV	VTA	SNr	SNc	CA1	CA2	CA3	DG
Sham/SAL	252.8±36.36	360.6±14.93	169.9±43.72	50.8±9.89	88.0±17.99	90.7±11.97	70.3±8.89	156.8±18.69	100.7±8.63
6-OHDA/SAL	247.1±17.22	375.6±45.40	153.8±40.00	48.4±8.12	104.9±23.07	78.9±9.36	84.0±9.85	146.4±16.42	92.6±4.38
Sham/PPX	368.2±18.16 **	424.9±28.29	233.8±54.70	63.3±16.17	113.0±20.95	108.5±13.55 *	132.1±26.82 *	211.8±10.65 **	135.3±13.11 **
6-OHDA/PPX	301.5±38.10 **	323.2±36.93	197.0±23.06	81.7±27.12	99.4±14.63	144.5±19.20 *	138.4±24.98 *	231.0±12.46 **	135.4±7.42 **

Brain region	LP	PV	LD	CL	MD	IMD	CM	PC	Po
Sham/SAL	75.2±11.20	566.9±39.36	59.1±2.68	126.8±36.48	85.5±3.80	121.8±20.24	63.5±7.55	53.5±7.18	74.9±11.22
6-OHDA/SAL	60.3±6.84	544.7±46.69	39.8±5.30	101.1±12.10	101.3±10.42	149.6±12.85	69.7±10.72	65.4±11.27	61.7±5.86
Sham/PPX	100.5±24.11	613.9±75.50	59.5±9.79	198.4±38.67 *	151.1±38.00	225.1±56.78 *	124.6±32.55	71.6±11.76	62.3±11.39
6-OHDA/PPX	96.1±12.08	714.0±91.96	45.4±7.04	153.6±21.61 *	117.1±24.01	211.3±35.35 *	93.7±15.70	44.2±12.88	50.4±9.15

Brain region	VPM	Rt	VPL	VM	STN	LH	PH	DMH	VMH
Sham/SAL	95.0±22.68	38.5±13.43	67.0±25.94	74.0±7.19	89.2±17.77	224.0±37.12	271.5±49.45	255.4±60.98	72.2±10.74
6-OHDA/SAL	65.4±11.38	23.8±5.15	39.1±7.25	75.6±9.35	100.9±63.69	212.3±16.17	270.3±60.00	303.4±37.22	84.0±13.68
Sham/PPX	100.9±16.32	83.2±33.62	69.3±11.62	96.4±19.30	76.8±18.21	206.7±11.04	262.9±40.70	276.8±67.75	103.1±17.20
6-OHDA/PPX	56.9±7.60	40.0±14.97	64.1±23.05	103.3±31.38	53.2±10.03	210.1±32.03	290.3±66.20	262.0±62.68	77.7±11.40

Table S2. Summary of statistical analyses.

Figure / Table	Panel	Number of Sample	Test used	Degrees of freedom and F/t/p value	Post-hoc test	Significance
Figure 2D		Sham = 6, 6-OHDA = 7	Student's t-test	t(11) = 10.00		Sham vs 6-OHDA, p < 0.01
Figure 2E	Disadvantageous choice	Sham / SAL = 6, 6-OHDA / SAL = 7	Two-way ANOVA	$F_{6\text{-OHDA}\,(1,24)}$ = 9.41, p < 0.01; $F_{PPX\,(1,24)}$ = 7.49, p < 0.05;	Bonferroni's multiple comparison	Sham / SAL vs 6-OHDA / SAL, p = 0.13
	Period1	Sham / PPX = 8, 6-OHDA / PPX = 7		$F_{6\text{-OHDA} \times PPX\,(1,24)}$ = 0.15, p = 0.70		Sham / SAL vs Sham / PPX, p = 0.19
						Sham / SAL vs 6-OHDA / PPX, p < 0.01
						6-OHDA / SAL vs 6-OHDA / PPX, p = 0.54
						Sham / PPX vs 6-OHDA / PPX, p = 0.30
	Disadvantageous choice	Sham / SAL = 6, 6-OHDA / SAL = 7	Two-way ANOVA	$F_{6\text{-OHDA}\,(1,24)}$ = 4.70, p < 0.05; $F_{PPX\,(1,24)}$ = 14.64, p < 0.01;	Bonferroni's multiple comparison	Sham / SAL vs 6-OHDA / SAL, p = 0.24
	Period2	Sham / PPX = 8, 6-OHDA / PPX = 7		$F_{6\text{-OHDA} \times PPX\,(1,24)}$ = 0.77, p = 0.39		Sham / SAL vs Sham / PPX, p < 0.05
						Sham / SAL vs 6-OHDA / PPX, p < 0.01
						6-OHDA / SAL vs 6-OHDA / PPX, p = 0.23
						Sham / PPX vs 6-OHDA / PPX, p > 0.99
Figure 2F	Response to P1	Sham / SAL = 6, 6-OHDA / SAL = 7	Two-way ANOVA	$F_{6\text{-OHDA}\,(1,24)}$ = 0.91, p = 0.35; $F_{PPX\,(1,24)}$ = 0.29, p = 0.59;	Not applicable	
	Period 1	Sham / PPX = 8, 6-OHDA / PPX = 7		$F_{6\text{-OHDA} \times PPX\,(1,24)}$ = 1.56, p = 0.22		
	Response to P1	Sham / SAL = 6, 6-OHDA / SAL = 7	Two-way ANOVA	$F_{6\text{-OHDA}\,(1,24)}$ = 2.54, p = 0.12; $F_{PPX\,(1,24)}$ = 0.41, p = 0.53;	Not applicable	
	Period 2	Sham / PPX = 8, 6-OHDA / PPX = 7		$F_{6\text{-OHDA} \times PPX\,(1,24)}$ = 2.02, p = 0.17		
Figure 2G	Response to P2	Sham / SAL = 6, 6-OHDA / SAL = 7	Two-way ANOVA	$F_{6\text{-OHDA}\,(1,24)}$ = 6.35, p < 0.05; $F_{PPX\,(1,24)}$ = 1.07, p = 0.31;	Bonferroni's multiple comparison	Sham / SAL vs 6-OHDA / SAL, p = 0.08
	Period 1	Sham / PPX = 8, 6-OHDA / PPX = 7		$F_{6\text{-OHDA} \times PPX\,(1,24)}$ = 1.63, p = 0.21		Sham / SAL vs Sham / PPX, p = 0.59
						Sham / SAL vs 6-OHDA / PPX, p = 0.12
						6-OHDA / SAL vs 6-OHDA / PPX, p > 0.99
						Sham / PPX vs 6-OHDA / PPX, p > 0.99
	Response to P2	Sham / SAL = 6, 6-OHDA / SAL = 7	Two-way ANOVA	$F_{6\text{-OHDA}\,(1,24)}$ = 6.80, p < 0.05; $F_{PPX\,(1,24)}$ = 2.47, p = 0.13;	Bonferroni's multiple comparison	Sham / SAL vs 6-OHDA / SAL, p < 0.05
	Period 2	Sham / PPX = 8, 6-OHDA / PPX = 7		$F_{6\text{-OHDA} \times PPX\,(1,24)}$ = 2.84, p = 0.11		Sham / SAL vs Sham / PPX, p = 0.16
						Sham / SAL vs 6-OHDA / PPX, p < 0.05
						6-OHDA / SAL vs 6-OHDA / PPX, p > 0.99
						Sham / PPX vs 6-OHDA / PPX, p > 0.99
Figure 2H	Response to P3	Sham / SAL = 6, 6-OHDA / SAL = 7	Two-way ANOVA	$F_{6\text{-OHDA}\,(1,24)}$ = 1.41, p = 0.25; $F_{PPX\,(1,24)}$ = 1.39, p = 0.25;	Not applicable	
	Period 1	Sham / PPX = 8, 6-OHDA / PPX = 7		$F_{6\text{-OHDA} \times PPX\,(1,24)}$ = 1.52, p = 0.23		
	Response to P3	Sham / SAL = 6, 6-OHDA / SAL = 7	Two-way ANOVA	$F_{6\text{-OHDA}\,(1,24)}$ = 1.08, p = 0.31; $F_{PPX\,(1,24)}$ = 7.85, p < 0.01;	Bonferroni's multiple comparison	Sham / SAL vs 6-OHDA / SAL, p = 0.50
	Period 2	Sham / PPX = 8, 6-OHDA / PPX = 7		$F_{6\text{-OHDA} \times PPX\,(1,24)}$ = 2.16, p = 0.15		Sham / SAL vs Sham / PPX, p < 0.05
						Sham / SAL vs 6-OHDA / PPX, p = 0.07
						6-OHDA / SAL vs 6-OHDA / PPX, p > 0.99
						Sham / PPX vs 6-OHDA / PPX, p > 0.99
Figure 2I	Response to P4	Sham / SAL = 6, 6-OHDA / SAL = 7	Two-way ANOVA	$F_{6\text{-OHDA}\,(1,24)}$ = 11.66, p < 0.01; $F_{PPX\,(1,24)}$ = 7.84, p < 0.01;	Bonferroni's multiple comparison	Sham / SAL vs 6-OHDA / SAL, p = 0.98
	Period 1	Sham / PPX = 8, 6-OHDA / PPX = 7		$F_{6\text{-OHDA} \times PPX\,(1,24)}$ = 2.15, p = 0.16		Sham / SAL vs Sham / PPX, p > 0.99
						Sham / SAL vs 6-OHDA / PPX, p < 0.01
						6-OHDA / SAL vs 6-OHDA / PPX, p < 0.05
						Sham / PPX vs 6-OHDA / PPX, p < 0.01
	Response to P4	Sham / SAL = 6, 6-OHDA / SAL = 7	Two-way ANOVA	$F_{6\text{-OHDA}\,(1,24)}$ = 6.88, p < 0.05; $F_{PPX\,(1,24)}$ = 7.39, p < 0.05;	Bonferroni's multiple comparison	Sham / SAL vs 6-OHDA / SAL, p > 0.99
	Period 2	Sham / PPX = 8, 6-OHDA / PPX = 7		$F_{6\text{-OHDA} \times PPX\,(1,24)}$ = 1.08, p = 0.31		Sham / SAL vs Sham / PPX, p > 0.99
						Sham / SAL vs 6-OHDA / PPX, p < 0.01
						6-OHDA / SAL vs 6-OHDA / PPX, p = 0.07
						Sham / PPX vs 6-OHDA / PPX, p = 0.06

Figure 2J	Total trials initiation<br>Period 1	Sham / SAL = 6, 6-OHDA / SAL = 7<br>Sham / PPX = 8, 6-OHDA / PPX = 7	Two-way ANOVA	$F_{\text{6-OHDA (1,24)}}$ = 12.88, p < 0.01; $F_{\text{PPX (1,24)}}$ = 0.41, p = 0.53;<br>$F_{\text{6-OHDA × PPX (1,24)}}$ = 0.11, p = 0.75	Bonferroni's multiple comparison	Sham / SAL vs 6-OHDA / SAL, p = 0.07<br>Sham / SAL vs Sham / PPX, p > 0.99<br>Sham / SAL vs 6-OHDA / PPX, p < 0.05<br>6-OHDA / SAL vs 6-OHDA / PPX, p > 0.99<br>Sham / PPX vs 6-OHDA / PPX, p = 0.12
	Total trials initiation<br>Period 2	Sham / SAL = 6, 6-OHDA / SAL = 7<br>Sham / PPX = 8, 6-OHDA / PPX = 7	Two-way ANOVA	$F_{\text{6-OHDA (1,24)}}$ = 15.90, p < 0.01; $F_{\text{PPX (1,24)}}$ = 0.05, p = 0.83;<br>$F_{\text{6-OHDA × PPX (1,24)}}$ = 1.75, p = 0.20	Bonferroni's multiple comparison	Sham / SAL vs 6-OHDA / SAL, p < 0.01<br>Sham / SAL vs Sham / PPX, p > 0.99<br>Sham / SAL vs 6-OHDA / PPX, p = 0.08<br>6-OHDA / SAL vs 6-OHDA / PPX, p > 0.99<br>Sham / PPX vs 6-OHDA / PPX, p = 0.31
Figure 2K	Premature response<br>Period 1	Sham / SAL = 6, 6-OHDA / SAL = 7<br>Sham / PPX = 8, 6-OHDA / PPX = 7	Two-way ANOVA	$F_{\text{6-OHDA (1,24)}}$ = 0.02, p = 0.90; $F_{\text{PPX (1,24)}}$ = 0.12, p = 0.73;<br>$F_{\text{6-OHDA × PPX (1,24)}}$ = 0.65, p = 0.43	Not applicable	
	Premature response<br>Period 2		Two-way ANOVA	$F_{\text{6-OHDA (1,24)}}$ = 0.05, p = 0.83; $F_{\text{PPX (1,24)}}$ = 9.50, p < 0.01;<br>$F_{\text{6-OHDA × PPX (1,24)}}$ = 1.19, p = 0.29	Bonferroni's multiple comparison	Sham / SAL vs 6-OHDA / SAL, p > 0.99<br>Sham / SAL vs Sham / PPX, p < 0.05<br>Sham / SAL vs 6-OHDA / PPX, p = 0.31<br>6-OHDA / SAL vs 6-OHDA / PPX, p = 0.85<br>Sham / PPX vs 6-OHDA / PPX, p > 0.99
Figure 2L	Omission<br>Period 1	Sham / SAL = 6, 6-OHDA / SAL = 7<br>Sham / PPX = 8, 6-OHDA / PPX = 7	Two-way ANOVA	$F_{\text{6-OHDA (1,24)}}$ = 8.96, p < 0.01; $F_{\text{PPX (1,24)}}$ = 13.23, p < 0.01;<br>$F_{\text{6-OHDA × PPX (1,24)}}$ = 0.16, p = 0.69	Bonferroni's multiple comparison	Sham / SAL vs 6-OHDA / SAL, p = 0.45<br>Sham / SAL vs Sham / PPX, p = 0.16<br>Sham / SAL vs 6-OHDA / PPX, p < 0.01<br>6-OHDA / SAL vs 6-OHDA / PPX, p < 0.05<br>Sham / PPX vs 6-OHDA / PPX, p = 0.10
	Omission<br>Period 2	Sham / SAL = 6, 6-OHDA / SAL = 7<br>Sham / PPX = 8, 6-OHDA / PPX = 7	Two-way ANOVA	$F_{\text{6-OHDA (1,24)}}$ = 8.34, p < 0.01; $F_{\text{PPX (1,24)}}$ = 0.38, p = 0.54;<br>$F_{\text{6-OHDA × PPX (1,24)}}$ = 0.19, p = 0.66	Bonferroni's multiple comparison	Sham / SAL vs 6-OHDA / SAL, p = 0.16<br>Sham / SAL vs Sham / PPX, p > 0.99<br>Sham / SAL vs 6-OHDA / PPX, p = 0.12<br>6-OHDA / SAL vs 6-OHDA / PPX, p > 0.99<br>Sham / PPX vs 6-OHDA / PPX, p = 0.43
Figure 2M	Collect latency<br>Period 1	Sham / SAL = 6, 6-OHDA / SAL = 7<br>Sham / PPX = 8, 6-OHDA / PPX = 7	Two-way ANOVA	$F_{\text{6-OHDA (1,24)}}$ = 2.94, p = 0.10; $F_{\text{PPX (1,24)}}$ = 0.78, p = 0.39;<br>$F_{\text{6-OHDA × PPX (1,24)}}$ = 0.73, p = 0.40	Not applicable	
	Collect latency<br>Period 2	Sham / SAL = 6, 6-OHDA / SAL = 7<br>Sham / PPX = 8, 6-OHDA / PPX = 7	Two-way ANOVA	$F_{\text{6-OHDA (1,24)}}$ = 6.42, p < 0.05; $F_{\text{PPX (1,24)}}$ = 2.78, p = 0.11;<br>$F_{\text{6-OHDA × PPX (1,24)}}$ = 1.57, p = 0.22	Bonferroni's multiple comparison	Sham / SAL vs 6-OHDA / SAL, p = 0.08<br>Sham / SAL vs Sham / PPX, p > 0.99<br>Sham / SAL vs 6-OHDA / PPX, p > 0.99<br>6-OHDA / SAL vs 6-OHDA / PPX, p = 0.24<br>Sham / PPX vs 6-OHDA / PPX, p > 0.99
Figure 3B	Disadvantageouse choice<br>Sham	PPX (0 mg/kg) = 5, PPX (0.01 mg/kg) = 6<br>PPX (0,1 mg/kg) = 6, PPX (0.3 mg/kg) = 6	One-way ANOVA	$F_{(3,19)}$ = 6.24, p < 0.01	Dunnett's multiple comparison	PPX (0 mg/kg) vs PPX (0.01 mg/kg), p = 0.62<br>PPX (0 mg/kg) vs PPX (0.1 mg/kg), p > 0.99<br>PPX (0 mg/kg) vs PPX (0.3 mg/kg), p < 0.05
	Disadvantageouse choice<br>6-OHDA	PPX (0 mg/kg) = 9, PPX (0.01 mg/kg) = 9<br>PPX (0,1 mg/kg) = 9, PPX (0.3 mg/kg) = 9	One-way ANOVA	$F_{(3,32)}$ = 5.24, p < 0.01	Dunnett's multiple comparison	PPX (0 mg/kg) vs PPX (0.01 mg/kg), p = 0.95<br>PPX (0 mg/kg) vs PPX (0.1 mg/kg), p > 0.99<br>PPX (0 mg/kg) vs PPX (0.3 mg/kg), p < 0.01
Figure 3D	Disadvantageouse choice	Sham-SAL / Vehicle = 4, Sham-SAL / PG-01037 = 4<br>6-OHDA-PPX / Vehicle = 4, 6-OHDA-PPX / PG-01037 = 5	Two-way ANOVA	$F_{\text{6-OHDA-PPX (1,13)}}$ = 41.21, p < 0.01; $F_{\text{PG-01037 (1,13)}}$ = 11.08, p < 0.01;<br>$F_{\text{6-OHDA-PPX × PG-01037 (1,13)}}$ = 9.05, p < 0.05	Bonferroni's multiple comparison	Sham-SAL / Vehicle vs Sham-SAL / PG-01037, p > 0.99<br>Sham-SAL / Vehicle vs 6-OHDA-PPX / Vehicle, p < 0.01<br>Sham-SAL / Vehicle vs 6-OHDA-PPX / PG-01037, p = 0.17<br>6-OHDA-PPX / Vehicle vs 6-OHDA-PPX / PG-01037, p < 0.01
Figure 4B	Number of c-Fos positive cells<br>STR	Sham / SAL = 8, 6-OHDA / SAL = 5<br>Sham / PPX = 5, 6-OHDA / PPX = 5	Two-way ANOVA	$F_{\text{6-OHDA (1,19)}}$ = 0.70, p = 0.41; $F_{\text{PPX (1,19)}}$ = 13.94, p < 0.01;<br>$F_{\text{6-OHDA × PPX (1,19)}}$ = 0.01, p = 0.93	Bonferroni's multiple comparison	Sham / SAL vs 6-OHDA / SAL, p > 0.99<br>Sham / SAL vs Sham / PPX, p = 0.07<br>Sham / SAL vs 6-OHDA / PPX, p < 0.05<br>6-OHDA / SAL vs 6-OHDA / PPX, p = 0.09<br>Sham / PPX vs 6-OHDA / PPX, p > 0.99
Figure 4D	Number of c-Fos positive cells<br>GPe	Sham / SAL = 8, 6-OHDA / SAL = 5<br>Sham / PPX = 5, 6-OHDA / PPX = 5	Two-way ANOVA	$F_{\text{6-OHDA (1,19)}}$ = 0.001, p = 0.98; $F_{\text{PPX (1,19)}}$ = 14.80, p < 0.01;<br>$F_{\text{6-OHDA × PPX (1,19)}}$ = 0.01, p = 0.93	Bonferroni's multiple comparison	Sham / SAL vs 6-OHDA / SAL, p > 0.99<br>Sham / SAL vs Sham / PPX, p < 0.05<br>Sham / SAL vs 6-OHDA / PPX, p < 0.05<br>6-OHDA / SAL vs 6-OHDA / PPX, p = 0.10<br>Sham / PPX vs 6-OHDA / PPX, p > 0.99
Figure 5E	mCherry+/c-Fos+	6-OHDA-PPX / Vehicle = 6, 6-OHDA-PPX / SALB = 6	Mann-Whitney U-test	U = 0		6-OHDA-PPX / Vehicle vs 6-OHDA-PPX / SALB, p < 0.01
Figure 5F	Disadvantageouse choice	Sham-SAL / Vehicle = 6, Sham-SAL / SALB = 5<br>6-OHDA-PPX / Vehicle = 7, 6-OHDA-PPX / SALB = 7	Two-way ANOVA	$F_{\text{6-OHDA-PPX (1,21)}}$ = 20.53, p < 0.01; $F_{\text{SALB (1,21)}}$ = 4.66, p < 0.05;<br>$F_{\text{6-OHDA-PPX × SALB (1,21)}}$ = 7.74, p < 0.05	Bonferroni's multiple comparison	Sham-SAL / Vehicle vs Sham-SAL / SALB, p > 0.99<br>Sham-SAL / Vehicle vs 6-OHDA-PPX / Vehicle, p < 0.01<br>Sham-SAL / Vehicle vs 6-OHDA-PPX / SALB, p = 0.40<br>6-OHDA-PPX / Vehicle vs 6-OHDA-PPX / SALB, p < 0.01
Figure S1	D3R mRNA	Sham / SAL = 8, 6-OHDA / SAL = 8<br>Sham / PPX = 8, 6-OHDA / PPX = 8	Two-way ANOVA	$F_{\text{6-OHDA (1,28)}}$ = 0.30, p = 0.59; $F_{\text{PPX (1,28)}}$ = 29.07, p < 0.01;<br>$F_{\text{6-OHDA × PPX (1,28)}}$ = 0.02, p = 0.88	Bonferroni's multiple comparison	Sham / SAL vs 6-OHDA / SAL, p > 0.99<br>Sham / SAL vs Sham / PPX, p < 0.01<br>Sham / SAL vs 6-OHDA / PPX, p < 0.01<br>6-OHDA / SAL vs 6-OHDA / PPX, p < 0.01<br>Sham / PPX vs 6-OHDA / PPX, p > 0.99
Figure S2D	Disadvantageouse choice	Sham-SAL / Vehicle = 4, Sham-SAL / SALB = 4<br>6-OHDA-PPX / Vehicle = 5, 6-OHDA-PPX / SALB = 5	Two-way ANOVA	$F_{\text{6-OHDA-PPX (1,14)}}$ = 12.61, p < 0.01; $F_{\text{SALB (1,14)}}$ = 0.06, p = 0.81;<br>$F_{\text{6-OHDA-PPX × SALB (1,14)}}$ = 0.45, p = 0.51	Bonferroni's multiple comparison	Sham-SAL / Vehicle vs Sham-SAL / SALB, p > 0.99<br>Sham-SAL / Vehicle vs 6-OHDA-PPX / Vehicle, p < 0.05<br>Sham-SAL / Vehicle vs 6-OHDA-PPX / SALB, p = 0.14<br>6-OHDA-PPX / Vehicle vs 6-OHDA-PPX / SALB, p > 0.99
Table S1	Number of c-Fos positive cells<br>Cg	Sham / SAL = 4, 6-OHDA / SAL = 5<br>Sham / PPX = 5, 6-OHDA / PPX = 5	Two-way ANOVA	$F_{\text{6-OHDA (1,15)}}$ = 2.49, p = 0.14; $F_{\text{PPX (1,15)}}$ = 0.09, p = 0.76;<br>$F_{\text{6-OHDA × PPX (1,15)}}$ = 0.17, p = 0.69	Not applicable	
	Number of c-Fos positive cells<br>PrL	Sham / SAL = 4, 6-OHDA / SAL = 5<br>Sham / PPX = 5, 6-OHDA / PPX = 5	Two-way ANOVA	$F_{\text{6-OHDA (1,15)}}$ = 1.87, p = 0.19; $F_{\text{PPX (1,15)}}$ = 0.18, p = 0.68;<br>$F_{\text{6-OHDA × PPX (1,15)}}$ = 0.04, p = 0.85	Not applicable	
	Number of c-Fos positive cells<br>IL	Sham / SAL = 4, 6-OHDA / SAL = 5<br>Sham / PPX = 5, 6-OHDA / PPX = 5	Two-way ANOVA	$F_{\text{6-OHDA (1,15)}}$ = 0.46, p = 0.51; $F_{\text{PPX (1,15)}}$ = 0.01, p = 0.92;<br>$F_{\text{6-OHDA × PPX (1,15)}}$ = 0.01, p = 0.93	Not applicable	
	Number of c-Fos positive cells<br>MO	Sham / SAL = 4, 6-OHDA / SAL = 5<br>Sham / PPX = 5, 6-OHDA / PPX = 5	Two-way ANOVA	$F_{\text{6-OHDA (1,13)}}$ = 1.40, p = 0.26; $F_{\text{PPX (1,13)}}$ = 0.91, p = 0.36;<br>$F_{\text{6-OHDA × PPX (1,13)}}$ = 0.0005, p = 0.98	Not applicable	
	Number of c-Fos positive cells<br>VO	Sham / SAL = 4, 6-OHDA / SAL = 5<br>Sham / PPX = 5, 6-OHDA / PPX = 5	Two-way ANOVA	$F_{\text{6-OHDA (1,14)}}$ = 0.52, p = 0.48; $F_{\text{PPX (1,14)}}$ = 0.36, p = 0.56;<br>$F_{\text{6-OHDA × PPX (1,14)}}$ = 0.04, p = 0.85	Not applicable	
	Number of c-Fos positive cells<br>LO	Sham / SAL = 4, 6-OHDA / SAL = 5<br>Sham / PPX = 5, 6-OHDA / PPX = 5	Two-way ANOVA	$F_{\text{6-OHDA (1,15)}}$ = 0.16, p = 0.69; $F_{\text{PPX (1,15)}}$ = 0.02, p = 0.88;<br>$F_{\text{6-OHDA × PPX (1,15)}}$ = 0.08, p = 0.78	Not applicable	
	Number of c-Fos positive cells<br>NAc	Sham / SAL = 4, 6-OHDA / SAL = 5<br>Sham / PPX = 5, 6-OHDA / PPX = 5	Two-way ANOVA	$F_{\text{6-OHDA (1,15)}}$ = 0.25, p = 0.63; $F_{\text{PPX (1,15)}}$ = 5.27, p < 0.05;<br>$F_{\text{6-OHDA × PPX (1,15)}}$ = 2.69, p = 0.12	Bonferroni's multiple comparison	Sham / SAL vs 6-OHDA / SAL, p = 0.81<br>Sham / SAL vs Sham / PPX, p > 0.99<br>Sham / SAL vs 6-OHDA / PPX, p > 0.99<br>6-OHDA / SAL vs 6-OHDA / PPX, p = 0.06<br>Sham / PPX vs 6-OHDA / PPX, p > 0.99
	Number of c-Fos positive cells<br>VP	Sham / SAL = 4, 6-OHDA / SAL = 5<br>Sham / PPX = 5, 6-OHDA / PPX = 5	Two-way ANOVA	$F_{\text{6-OHDA (1,15)}}$ = 0.11, p = 0.74; $F_{\text{PPX (1,15)}}$ = 4.22, p = 0.06;<br>$F_{\text{6-OHDA × PPX (1,15)}}$ = 3.10, p = 0.10	Not applicable	
	Number of c-Fos positive cells<br>LSD	Sham / SAL = 4, 6-OHDA / SAL = 5<br>Sham / PPX = 5, 6-OHDA / PPX = 5	Two-way ANOVA	$F_{\text{6-OHDA (1,11)}}$ = 0.47, p = 0.51; $F_{\text{PPX (1,11)}}$ = 2.21, p = 0.16;<br>$F_{\text{6-OHDA × PPX (1,11)}}$ = 0.0002, p = 0.99	Not applicable	
	Number of c-Fos positive cells<br>LSI	Sham / SAL = 4, 6-OHDA / SAL = 5<br>Sham / PPX = 5, 6-OHDA / PPX = 5	Two-way ANOVA	$F_{\text{6-OHDA (1,15)}}$ = 1.61, p = 0.22; $F_{\text{PPX (1,15)}}$ = 8.85, p < 0.01;<br>$F_{\text{6-OHDA × PPX (1,15)}}$ = 1.14, p = 0.30	Bonferroni's multiple comparison	Sham / SAL vs 6-OHDA / SAL, p > 0.99<br>Sham / SAL vs Sham / PPX, p = 0.07<br>Sham / SAL vs 6-OHDA / PPX, p > 0.99<br>6-OHDA / SAL vs 6-OHDA / PPX, p = 0.93<br>Sham / PPX vs 6-OHDA / PPX, p = 0.54
	Number of c-Fos positive cells<br>LSV	Sham / SAL = 4, 6-OHDA / SAL = 5<br>Sham / PPX = 5, 6-OHDA / PPX = 5	Two-way ANOVA	$F_{\text{6-OHDA (1,15)}}$ = 1.52, p = 0.24; $F_{\text{PPX (1,15)}}$ = 0.03, p = 0.87;<br>$F_{\text{6-OHDA × PPX (1,15)}}$ = 2.76, p = 0.12	Not applicable	
	Number of c-Fos positive cells<br>VTA	Sham / SAL = 4, 6-OHDA / SAL = 5<br>Sham / PPX = 5, 6-OHDA / PPX = 5	Two-way ANOVA	$F_{\text{6-OHDA (1,15)}}$ = 0.39, p = 0.54; $F_{\text{PPX (1,15)}}$ = 1.62, p = 0.22;<br>$F_{\text{6-OHDA × PPX (1,15)}}$ = 0.06, p = 0.81	Not applicable	
	Number of c-Fos positive cells<br>SNr	Sham / SAL = 4, 6-OHDA / SAL = 5<br>Sham / PPX = 5, 6-OHDA / PPX = 5	Two-way ANOVA	$F_{\text{6-OHDA (1,15)}}$ = 0.20, p = 0.66; $F_{\text{PPX (1,15)}}$ = 1.65, p = 0.22;<br>$F_{\text{6-OHDA × PPX (1,15)}}$ = 0.34, p = 0.57	Not applicable	
	Number of c-Fos positive cells<br>SNc	Sham / SAL = 4, 6-OHDA / SAL = 5<br>Sham / PPX = 5, 6-OHDA / PPX = 5	Two-way ANOVA	$F_{\text{6-OHDA (1,15)}}$ = 0.01, p = 0.94; $F_{\text{PPX (1,15)}}$ = 0.24, p = 0.63;<br>$F_{\text{6-OHDA × PPX (1,15)}}$ = 0.59, p = 0.45	Not applicable	
	Number of c-Fos positive cells<br>CA1	Sham / SAL = 4, 6-OHDA / SAL = 5<br>Sham / PPX = 5, 6-OHDA / PPX = 5	Two-way ANOVA	$F_{\text{6-OHDA (1,15)}}$ = 0.71, p = 0.41; $F_{\text{PPX (1,15)}}$ = 8.47, p < 0.05;<br>$F_{\text{6-OHDA × PPX (1,15)}}$ = 2.77, p = 0.12	Bonferroni's multiple comparison	Sham / SAL vs 6-OHDA / SAL, p > 0.99<br>Sham / SAL vs Sham / PPX, p > 0.99<br>Sham / SAL vs 6-OHDA / PPX, p = 0.10<br>6-OHDA / SAL vs 6-OHDA / PPX, p < 0.05<br>Sham / PPX vs 6-OHDA / PPX, p = 0.44
	Number of c-Fos positive cells<br>CA2	Sham / SAL = 4, 6-OHDA / SAL = 5<br>Sham / PPX = 5, 6-OHDA / PPX = 5	Two-way ANOVA	$F_{\text{6-OHDA (1,15)}}$ = 0.24, p = 0.63; $F_{\text{PPX (1,15)}}$ = 8.00, p < 0.05;<br>$F_{\text{6-OHDA × PPX (1,15)}}$ = 0.03, p = 0.86	Bonferroni's multiple comparison	Sham / SAL vs 6-OHDA / SAL, p > 0.99<br>Sham / SAL vs Sham / PPX, p = 0.28<br>Sham / SAL vs 6-OHDA / PPX, p = 0.19<br>6-OHDA / SAL vs 6-OHDA / PPX, p = 0.36<br>Sham / PPX vs 6-OHDA / PPX, p > 0.99
	Number of c-Fos positive cells<br>CA3	Sham / SAL = 4, 6-OHDA / SAL = 5<br>Sham / PPX = 5, 6-OHDA / PPX = 5	Two-way ANOVA	$F_{\text{6-OHDA (1,15)}}$ = 0.09, p = 0.77; $F_{\text{PPX (1,15)}}$ = 22.99, p < 0.01;<br>$F_{\text{6-OHDA × PPX (1,15)}}$ = 1.03, p = 0.33	Bonferroni's multiple comparison	Sham / SAL vs 6-OHDA / SAL, p > 0.99<br>Sham / SAL vs Sham / PPX, p = 0.10<br>Sham / SAL vs 6-OHDA / PPX, p < 0.05<br>6-OHDA / SAL vs 6-OHDA / PPX, p < 0.01<br>Sham / PPX vs 6-OHDA / PPX, p > 0.99
	Number of c-Fos positive cells<br>DG	Sham / SAL = 4, 6-OHDA / SAL = 5<br>Sham / PPX = 5, 6-OHDA / PPX = 5	Two-way ANOVA	$F_{\text{6-OHDA (1,15)}}$ = 0.19, p = 0.67; $F_{\text{PPX (1,15)}}$ = 18.23, p < 0.01;<br>$F_{\text{6-OHDA × PPX (1,15)}}$ = 0.21, p = 0.66	Bonferroni's multiple comparison	Sham / SAL vs 6-OHDA / SAL, p > 0.99<br>Sham / SAL vs Sham / PPX, p = 0.10<br>Sham / SAL vs 6-OHDA / PPX, p = 0.09<br>6-OHDA / SAL vs 6-OHDA / PPX, p < 0.05<br>Sham / PPX vs 6-OHDA / PPX, p > 0.99
	Number of c-Fos positive cells<br>LP	Sham / SAL = 4, 6-OHDA / SAL = 5<br>Sham / PPX = 5, 6-OHDA / PPX = 5	Two-way ANOVA	$F_{\text{6-OHDA (1,15)}}$ = 0.38, p = 0.55; $F_{\text{PPX (1,15)}}$ = 3.87, p = 0.07;<br>$F_{\text{6-OHDA × PPX (1,15)}}$ = 0.11, p = 0.74	Not applicable	
	Number of c-Fos positive cells<br>PV	Sham / SAL = 4, 6-OHDA / SAL = 5<br>Sham / PPX = 5, 6-OHDA / PPX = 5	Two-way ANOVA	$F_{\text{6-OHDA (1,15)}}$ = 0.31, p = 0.59; $F_{\text{PPX (1,15)}}$ = 2.39, p = 0.14;<br>$F_{\text{6-OHDA × PPX (1,15)}}$ = 0.76, p = 0.40	Not applicable	
	Number of c-Fos positive cells<br>LD	Sham / SAL = 4, 6-OHDA / SAL = 5<br>Sham / PPX = 5, 6-OHDA / PPX = 5	Two-way ANOVA	$F_{\text{6-OHDA (1,15)}}$ = 5.53, p < 0.05; $F_{\text{PPX (1,15)}}$ = 0.18, p = 0.67;<br>$F_{\text{6-OHDA × PPX (1,15)}}$ = 0.14, p = 0.72	Bonferroni's multiple comparison	Sham / SAL vs 6-OHDA / SAL, p = 0.41<br>Sham / SAL vs Sham / PPX, p > 0.99<br>Sham / SAL vs 6-OHDA / PPX, p > 0.99<br>6-OHDA / SAL vs 6-OHDA / PPX, p > 0.99<br>Sham / PPX vs 6-OHDA / PPX, p = 0.84

	Number of c-Fos positive cells	Sham / SAL = 4, 6-OHDA / SAL = 5	Two-way ANOVA	$F_{6\text{-OHDA}\,(1,15)} = 1.51$, $p = 0.24$; $F_{PPX\,(1,15)} = 4.68$, $p < 0.05$;	Bonferroni's multiple comparison	Sham / SAL vs 6-OHDA / SAL, p > 0.99
	CL	Sham / PPX = 5, 6-OHDA / PPX = 5		$F_{6\text{-OHDA} \times PPX\,(1,15)} = 0.11$, $p = 0.74$		Sham / SAL vs Sham / PPX, p = 0.53
						Sham / SAL vs 6-OHDA / PPX, p > 0.99
						6-OHDA / SAL vs 6-OHDA / PPX, p > 0.99
						Sham / PPX vs 6-OHDA / PPX, p > 0.99
	Number of c-Fos positive cells	Sham / SAL = 4, 6-OHDA / SAL = 5	Two-way ANOVA	$F_{6\text{-OHDA}\,(1,15)} = 0.14$, $p = 0.72$; $F_{PPX\,(1,15)} = 2.74$, $p = 0.12$;	Not applicable	
	MD	Sham / PPX = 5, 6-OHDA / PPX = 5		$F_{6\text{-OHDA} \times PPX\,(1,15)} = 1.02$, $p = 0.33$		
	Number of c-Fos positive cells	Sham / SAL = 4, 6-OHDA / SAL = 5	Two-way ANOVA	$F_{6\text{-OHDA}\,(1,15)} = 0.04$, $p = 0.85$; $F_{PPX\,(1,15)} = 4.92$, $p < 0.05$;	Bonferroni's multiple comparison	Sham / SAL vs 6-OHDA / SAL, p > 0.99
	IMD	Sham / PPX = 5, 6-OHDA / PPX = 5		$F_{6\text{-OHDA} \times PPX\,(1,15)} = 0.31$, $p = 0.58$		Sham / SAL vs Sham / PPX, p = 0.38
						Sham / SAL vs 6-OHDA / PPX, p = 0.60
						6-OHDA / SAL vs 6-OHDA / PPX, p > 0.99
						Sham / PPX vs 6-OHDA / PPX, p > 0.99
	Number of c-Fos positive cells	Sham / SAL = 4, 6-OHDA / SAL = 5	Two-way ANOVA	$F_{6\text{-OHDA}\,(1,15)} = 0.37$, $p = 0.55$; $F_{PPX\,(1,15)} = 4.39$, $p = 0.054$;	Not applicable	
	CM	Sham / PPX = 5, 6-OHDA / PPX = 5		$F_{6\text{-OHDA} \times PPX\,(1,15)} = 0.83$, $p = 0.38$		
	Number of c-Fos positive cells	Sham / SAL = 4, 6-OHDA / SAL = 5	Two-way ANOVA	$F_{6\text{-OHDA}\,(1,15)} = 0.45$, $p = 0.51$; $F_{PPX\,(1,15)} = 0.02$, $p = 0.90$;	Not applicable	
	PC	Sham / PPX = 5, 6-OHDA / PPX = 5		$F_{6\text{-OHDA} \times PPX\,(1,15)} = 2.96$, $p = 0.11$		
	Number of c-Fos positive cells	Sham / SAL = 4, 6-OHDA / SAL = 5	Two-way ANOVA	$F_{6\text{-OHDA}\,(1,15)} = 1.72$, $p = 0.21$; $F_{PPX\,(1,15)} = 1.56$, $p = 0.23$;	Not applicable	
	Po	Sham / PPX = 5, 6-OHDA / PPX = 5		$F_{6\text{-OHDA} \times PPX\,(1,15)} = 0.01$, $p = 0.94$		
	Number of c-Fos positive cells	Sham / SAL = 4, 6-OHDA / SAL = 5	Two-way ANOVA	$F_{6\text{-OHDA}\,(1,15)} = 6.26$, $p < 0.05$; $F_{PPX\,(1,15)} = 0.01$, $p = 0.93$;	Bonferroni's multiple comparison	Sham / SAL vs 6-OHDA / SAL, p = 0.94
	VPM	Sham / PPX = 5, 6-OHDA / PPX = 5		$F_{6\text{-OHDA} \times PPX\,(1,15)} = 0.24$, $p = 0.63$		Sham / SAL vs Sham / PPX, p > 0.99
						Sham / SAL vs 6-OHDA / PPX, p = 0.48
						6-OHDA / SAL vs 6-OHDA / PPX, p > 0.99
						Sham / PPX vs 6-OHDA / PPX, p = 0.23
	Number of c-Fos positive cells	Sham / SAL = 4, 6-OHDA / SAL = 5	Two-way ANOVA	$F_{6\text{-OHDA}\,(1,15)} = 1.99$, $p = 0.18$; $F_{PPX\,(1,15)} = 2.20$, $p = 0.16$;	Not applicable	
	Rt	Sham / PPX = 5, 6-OHDA / PPX = 5		$F_{6\text{-OHDA} \times PPX\,(1,15)} = 0.48$, $p = 0.50$		
	Number of c-Fos positive cells	Sham / SAL = 4, 6-OHDA / SAL = 5	Two-way ANOVA	$F_{6\text{-OHDA}\,(1,15)} = 0.86$, $p = 0.37$; $F_{PPX\,(1,15)} = 0.58$, $p = 0.46$;	Not applicable	
	VPL	Sham / PPX = 5, 6-OHDA / PPX = 5		$F_{6\text{-OHDA} \times PPX\,(1,15)} = 0.40$, $p = 0.53$		
	Number of c-Fos positive cells	Sham / SAL = 4, 6-OHDA / SAL = 5	Two-way ANOVA	$F_{6\text{-OHDA}\,(1,15)} = 0.04$, $p = 0.84$; $F_{PPX\,(1,15)} = 1.50$, $p = 0.24$;	Not applicable	
	VM	Sham / PPX = 5, 6-OHDA / PPX = 5		$F_{6\text{-OHDA} \times PPX\,(1,15)} = 0.02$, $p = 0.90$		
	Number of c-Fos positive cells	Sham / SAL = 4, 6-OHDA / SAL = 5	Two-way ANOVA	$F_{6\text{-OHDA}\,(1,15)} = 0.03$, $p = 0.87$; $F_{PPX\,(1,15)} = 0.68$, $p = 0.42$;	Not applicable	
	STN	Sham / PPX = 5, 6-OHDA / PPX = 5		$F_{6\text{-OHDA} \times PPX\,(1,15)} = 0.24$, $p = 0.63$		
	Number of c-Fos positive cells	Sham / SAL = 4, 6-OHDA / SAL = 5	Two-way ANOVA	$F_{6\text{-OHDA}\,(1,15)} = 0.03$, $p = 0.87$; $F_{PPX\,(1,15)} = 0.15$, $p = 0.71$;	Not applicable	
	LH	Sham / PPX = 5, 6-OHDA / PPX = 5		$F_{6\text{-OHDA} \times PPX\,(1,15)} = 0.09$, $p = 0.77$		
	Number of c-Fos positive cells	Sham / SAL = 4, 6-OHDA / SAL = 5	Two-way ANOVA	$F_{6\text{-OHDA}\,(1,15)} = 0.05$, $p = 0.82$; $F_{PPX\,(1,15)} = 0.01$, $p = 0.92$;	Not applicable	
	PH	Sham / PPX = 5, 6-OHDA / PPX = 5		$F_{6\text{-OHDA} \times PPX\,(1,15)} = 0.06$, $p = 0.81$		
	Number of c-Fos positive cells	Sham / SAL = 4, 6-OHDA / SAL = 5	Two-way ANOVA	$F_{6\text{-OHDA}\,(1,15)} = 0.08$, $p = 0.78$; $F_{PPX\,(1,15)} = 0.03$, $p = 0.87$;	Not applicable	
	DMH	Sham / PPX = 5, 6-OHDA / PPX = 5		$F_{6\text{-OHDA} \times PPX\,(1,15)} = 0.29$, $p = 0.60$		
	Number of c-Fos positive cells	Sham / SAL = 4, 6-OHDA / SAL = 5	Two-way ANOVA	$F_{6\text{-OHDA}\,(1,15)} = 0.24$, $p = 0.63$; $F_{PPX\,(1,15)} = 0.78$, $p = 0.39$;	Not applicable	
	VMH	Sham / PPX = 5, 6-OHDA / PPX = 5		$F_{6\text{-OHDA} \times PPX\,(1,15)} = 1.79$, $p = 0.20$		
